# Metabolic–Respiratory Priming in judo: a physiological perspective on performance onset

**DOI:** 10.3389/fspor.2026.1812920

**Published:** 2026-05-11

**Authors:** Francesco Bruyère, Federico Aimar, Emidio Centracchio, Andrea Ferretti

**Affiliations:** 1 Department of Life Sciences and Systems Biology, University of Turin, Turin, Italy; 2 Italian National Judo Team, FIJLKAM, Rome, Italy

**Keywords:** combat sports, judo, lactate metabolism, metabolic priming, oxygen deficit, PAPE, performance onset, VO_2_ kinetics

## Abstract

Combat sports impose a distinctive performance constraint: maximal tactical and metabolic demands emerge immediately at match onset, before oxidative metabolism has reached steady state. In judo, grip engagement, off-balancing attempts, and scoring actions frequently occur within the opening minute, creating a mismatch between energetic demand and metabolic readiness that may influence tactical control and match outcome. This Perspective formalizes Metabolic–Respiratory Priming (MRP) as a practitioner-derived, hypothesis-generating framework addressing early-match metabolic lag. Grounded in oxygen uptake (VO₂) kinetics and contemporary lactate metabolism concepts, MRP is defined as a brief terminal activation phase designed to accelerate VO₂ kinetics, reduce oxygen deficit, and align metabolic responses at performance onset. Although MRP may coexist with neuromuscular priming strategies such as post-activation performance enhancement (PAPE), it is conceptually distinct in objective, timing, and expression window. We outline the physiological rationale for MRP, propose applied implementation considerations under elite competition ecology, and present a representative warm-up architecture used within the Italian National Judo Team. Limitations and future research priorities are discussed to encourage empirical validation across intermittent combat sports characterized by abrupt high-intensity onset.

## 1 Introduction (revised)

Judo performance is often shaped early. The opening exchanges frequently determine grip dominance, positional initiative, and the probability of subsequent scoring sequences (1, 2, 20, 24). At the elite level, these interactions can be decisive, as opponents are tactically prepared, margins are small, and a single successful action may substantially influence bout trajectory.

**Figure 1:**
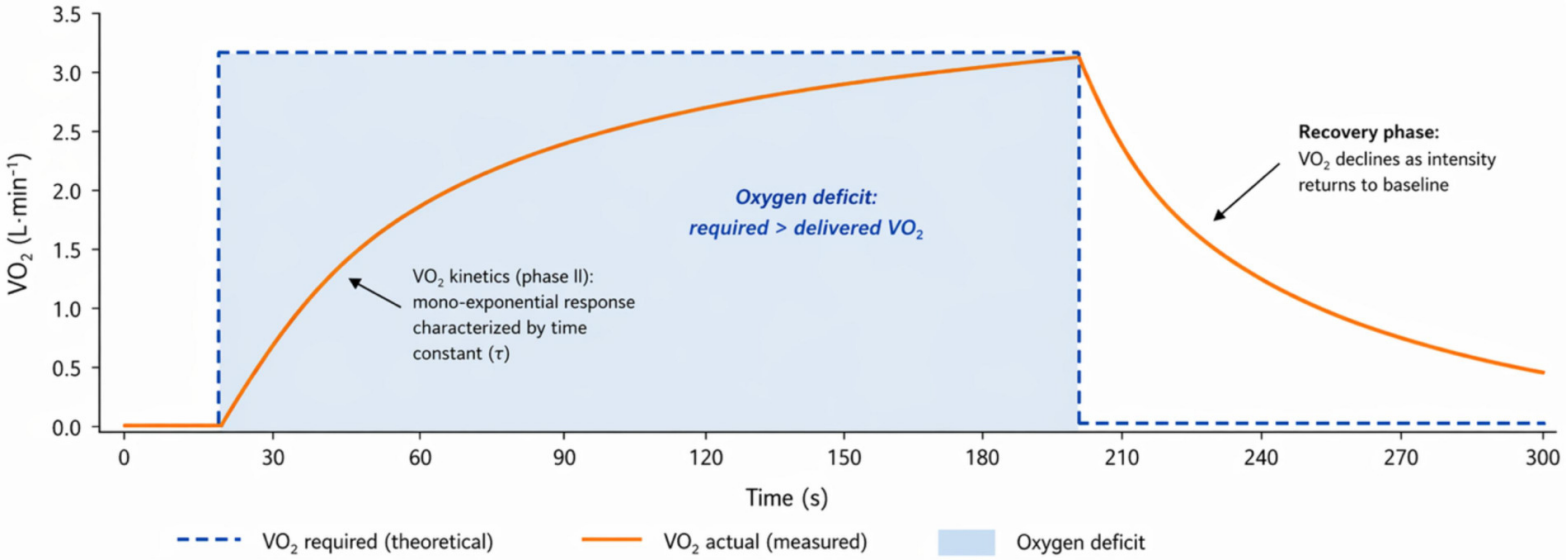
Oxygen deficit during abrupt onset of high-intensity exercise. The dashed line represents theoretical oxygen demand, while the solid line represents the pulmonary VO₂ response. The shaded area illustrates the oxygen deficit occurring before oxidative metabolism reaches steady state.

Unlike endurance disciplines, where intensity rises progressively, combat sports demand near-maximal mechanical output at match onset (2, 3). This creates a distinct physiological challenge: athletes must express explosive force, sustain high-intensity grappling, and execute rapid tactical decisions before metabolic systems have fully aligned with energetic demand. While judo research has described physiological profiles and time–motion characteristics, the constraint imposed by delayed metabolic readiness at exercise onset remains conceptually under-structured.

In high-performance environments, coaches have long employed a brief high-intensity activation phase within the warm-up to “break the breath” (in Italy, *rottura fiato*), referring to a subjective ventilatory and metabolic transition intended to mitigate early-bout perceptual shock and tempo mismatch. Despite widespread applied use, this practice lacks unified terminology and explicit physiological framing within the scientific literature (4, 5).

This Perspective introduces Metabolic–Respiratory Priming (MRP) to formalize this applied strategy as a hypothesis-generating conceptual framework, clarify its scope, and propose testable mechanisms relevant to elite judo competition.

## 2 Early-match metabolic lag in judo: why onset matters

Abrupt high-intensity exercise is characterized by a delay in pulmonary oxygen uptake (VO₂) relative to metabolic demand. VO₂ kinetics typically follow a mono-exponential trajectory requiring time to approach steady state, with the phase II time constant (*τ*) commonly on the order of ∼2–3 min in trained individuals, depending on exercise intensity and domain (6, 7). During this interval, the mismatch between required and delivered oxidative energy defines the oxygen deficit (7, 21, 25) (Figure 1).

To sustain external work while VO₂ lags, reliance on non-oxidative pathways increases: glycolytic flux rises, lactate accumulates, and perceived exertion escalates (8–10).

This mismatch is particularly consequential in judo, where early-bout actions are both mechanically intense and tactically decisive. Grip engagement, off-balancing attempts, explosive actions, and transitions frequently occur within the oxygen deficit window (2). Athletes are therefore required to operate at competition tempo before oxidative metabolism has fully aligned with energetic demand (7). In applied settings, this may manifest as transient respiratory disruption, disproportionate effort perception, and reduced motor fluency (8).

From a performance perspective, early-match metabolic lag is not merely a reflection of general aerobic fitness but a time-specific physiological constraint (18, 22, 23). Athletes may possess sufficient metabolic capacity to sustain later-bout intensity once VO₂ stabilizes, yet remain vulnerable during the opening exchanges when tactical initiative and positional control are established (2, 6). This framing supports the rationale for preparation strategies targeting the onset phase of performance.

## 3 Definition and scope of Metabolic–Respiratory Priming (MRP)

Within elite judo settings, a brief high-intensity activation phase is frequently employed at the end of warm-up to mitigate early-bout metabolic disruption. Although related concepts appear within the broader warm-up and priming literature, this practice in combat sports has rarely been formalized as a distinct construct explicitly linked to exercise onset physiology (4, 5).

We define Metabolic–Respiratory Priming (MRP) as a brief warm-up phase composed of intermittent high-intensity efforts designed to accelerate pulmonary oxygen uptake (VO₂) kinetics, reduce oxygen deficit, and enhance metabolic–respiratory readiness at performance onset (6, 7).

Although MRP involves high-intensity work, its defining feature is timing and metabolic objective, rather than intensity *per se*: preparing the athlete to operate at competition tempo from the initial contest actions.

Operationally, MRP is positioned after general and sport-specific warm-up activities and followed by a controlled recovery window leading into match call-up. The intended performance effects are expressed primarily during the opening phase of the bout, rather than later stages of competition. As a conceptual framework, MRP is proposed to generate testable hypotheses regarding metabolic–respiratory readiness at exercise onset.

### 3.1 Distinction from neuromuscular priming (PAP/PAPE)

MRP is conceptually distinct from post-activation potentiation (PAP) and post-activation performance enhancement (PAPE). PAP/PAPE primarily target neural and contractile mechanisms, such as increased motoneuron excitability and phosphorylation (19) of myosin regulatory light chains, leading to transient improvements in mechanical responsiveness and explosive force production (11–13).

By contrast, MRP targets metabolic–respiratory readiness at exercise onset. Its proposed objective is to initiate performance from an elevated VO₂ baseline and a pre-activated metabolic state, thereby reducing oxygen deficit magnitude and attenuating abrupt glycolytic perturbation during the initial exchanges (5, 7).

These strategies are not mutually exclusive. In applied settings, MRP and PAPE may be sequenced compatibly: MRP facilitates metabolic alignment earlier in the warm-up sequence, whereas a brief non-glycolytic neuromuscular stimulus may be applied closer to call-up to enhance explosive responsiveness without imposing additional metabolic load (12).

## 4 Physiological rationale: how MRP may work

Metabolic–Respiratory Priming (MRP) is proposed to address early-match metabolic lag through three interacting pathways: acceleration of pulmonary oxygen uptake (VO₂) kinetics, modulation of lactate dynamics, and improved metabolic–neuromuscular convergence at exercise onset (6, 7, 9).

### 4.1 Acceleration of VO₂ kinetics and reduction of oxygen deficit

Pulmonary VO₂ does not increase instantaneously at the onset of high-intensity exercise. Instead, it follows a mono-exponential trajectory characterized by the phase II time constant (*τ*), during which oxidative metabolism progressively adjusts to energetic demand (6, 7). When exercise intensity rises abruptly, the mismatch between required and delivered oxidative energy defines the oxygen deficit (7).

A prior high-intensity activation, as implemented in MRP, may elevate ventilation, cardiac output, and muscle oxygen utilization, enabling performance to begin from an increased VO₂ baseline (5). This shift is expected to reduce oxygen deficit magnitude and attenuate reliance on abrupt glycolytic compensation during the opening contest phase (14).

In applied settings, athletes and coaches frequently report reduced perceptual "shock" at onset and a smoother transition into early high-intensity exchanges. Although experiential, these observations are consistent with established relationships linking oxygen deficit, ventilatory strain, and perceived exertion during abrupt exercise transitions (7, 8).

The practical implication is time-specific: improved metabolic alignment at exercise onset may support early technical fluency, such as grip engagement, explosive entries, and off-balancing, precisely when tactical initiative is established.

### 4.2 Lactate dynamics as proactive metabolic organization

Contemporary models recognize lactate not solely as a fatigue-related byproduct but as a central metabolic intermediate within lactate shuttle systems linking glycolytic and oxidative tissues (9, 15, 16). Within this framework, lactate functions as both an energy substrate and a signaling molecule coordinating metabolic responses.

A controlled, transient elevation of lactate during MRP may facilitate early activation of transport and oxidation pathways, promoting a metabolic state in which lactate turnover is already engaged at performance onset (9). Rather than accumulating steeply as a reactive consequence of delayed oxidative contribution, lactate dynamics may exhibit a smoother trajectory during the initial exchanges.

When oxidative metabolism “arrives late,” early lactate rise may be sharper, contributing to rapid increases in perceived exertion and disturbances in motor coordination (8). By pre-activating metabolic turnover, MRP is hypothesized to attenuate this early perturbation, supporting coordination and effort regulation during the opening contest phase.

This interpretation does not imply reduced lactate production, but rather altered timing and integration of lactate appearance and utilization at exercise onset.

### 4.3 Metabolic–neuromuscular convergence at performance onset

Although MRP is not a neuromuscular potentiation strategy, metabolic readiness strongly influences how force is generated and sustained under high-intensity conditions. A large oxygen deficit increases the metabolic cost of force production, accelerates perceived exertion, and may disrupt motor control (10).

By reducing early metabolic disruption, MRP may lower the energetic and perceptual cost of expressing explosive and isometric actions at onset, enabling more effective coupling between tactical intent, neuromuscular execution, and metabolic support during the opening exchanges.

In elite contexts, metabolic alignment induced by MRP may be complemented by a brief post-activation performance enhancement (PAPE) stimulus applied shortly before call-up. Such stimuli, typically short, non-glycolytic explosive or isometric actions, may enhance neuromuscular responsiveness without imposing additional metabolic load (12, 13).

Crucially, the persistence of these priming effects is likely time-dependent and influenced by the balance between activation and recovery. While prior studies suggest a decay of priming effects within ∼15–20 min under passive conditions, intermittent activity and low-level reactivation during the pre-competition phase may contribute to maintaining metabolic readiness beyond passive recovery scenarios.

The conceptual emphasis is therefore convergence: metabolic readiness established earlier, neuromuscular sharpness refined later, both expressed at match onset.

## 5 Applied implementation considerations under competition ecology

Metabolic–Respiratory Priming (MRP) is not intended as a fixed protocol but as a scalable applied framework requiring calibration between metabolic activation and residual fatigue risk. Key variables include intensity, duration, and recovery window (6, 17).

### 5.1 Intensity and structure

In elite practice, MRP typically consists of intermittent high-intensity efforts designed to approximate competition-level breathing, cardiovascular activation, and metabolic turnover. The objective is to induce a meaningful metabolic–respiratory stimulus while avoiding prolonged continuous work that would shift responses toward steady-state aerobic exercise (5).

Applied anchors may include severe but transient perceived exertion and a clear ventilatory breakthrough. Common modalities in judo environments include high-intensity grip exchanges, short *uchi-komi* (repetitive entry drills) or *nage-komi* (repeated throwing sequences), and brief simulated contest actions performed intermittently to reflect contest dynamics (2).

Intermittency is central, allowing substantial metabolic activation while limiting excessive fatigue accumulation associated with longer continuous efforts (17).

### 5.2 Duration

Total work duration is brief by design, typically comprising only a few minutes of accumulated high-intensity activity. This duration is generally sufficient to activate metabolic–respiratory systems while preserving neuromuscular freshness and technical sharpness for bout onset (17).

Excessively prolonged activation phases may increase the likelihood of residual fatigue, elevated ventilatory strain, or perceptual overload.

### 5.3 Recovery window and decay dynamics

A recovery interval following MRP is required to allow ventilation, heart rate, and perceived exertion to stabilize while retaining priming effects (6, 7).

Importantly, the persistence of priming effects is time-dependent and influenced by the balance between activation and recovery. While prior literature suggests a decay of priming effects within ∼15–20 min under passive conditions, pre-competition contexts are rarely passive. Light activity, technical rehearsal, and intermittent reactivation may contribute to maintaining metabolic readiness beyond passive recovery scenarios.

In elite competition settings characterized by structured call-up procedures, a controlled recovery window is commonly implemented. When delays extend beyond the effective priming window, a brief re-activation block may be employed to restore metabolic readiness without inducing cumulative fatigue (17).

In youth or rapid-turnover tournament formats, reduced-load variants may be preferable to minimize the risk of entering competition with unresolved metabolic or ventilatory strain.

### 5.4 Representative sequencing (applied example)

**Figure 2:**
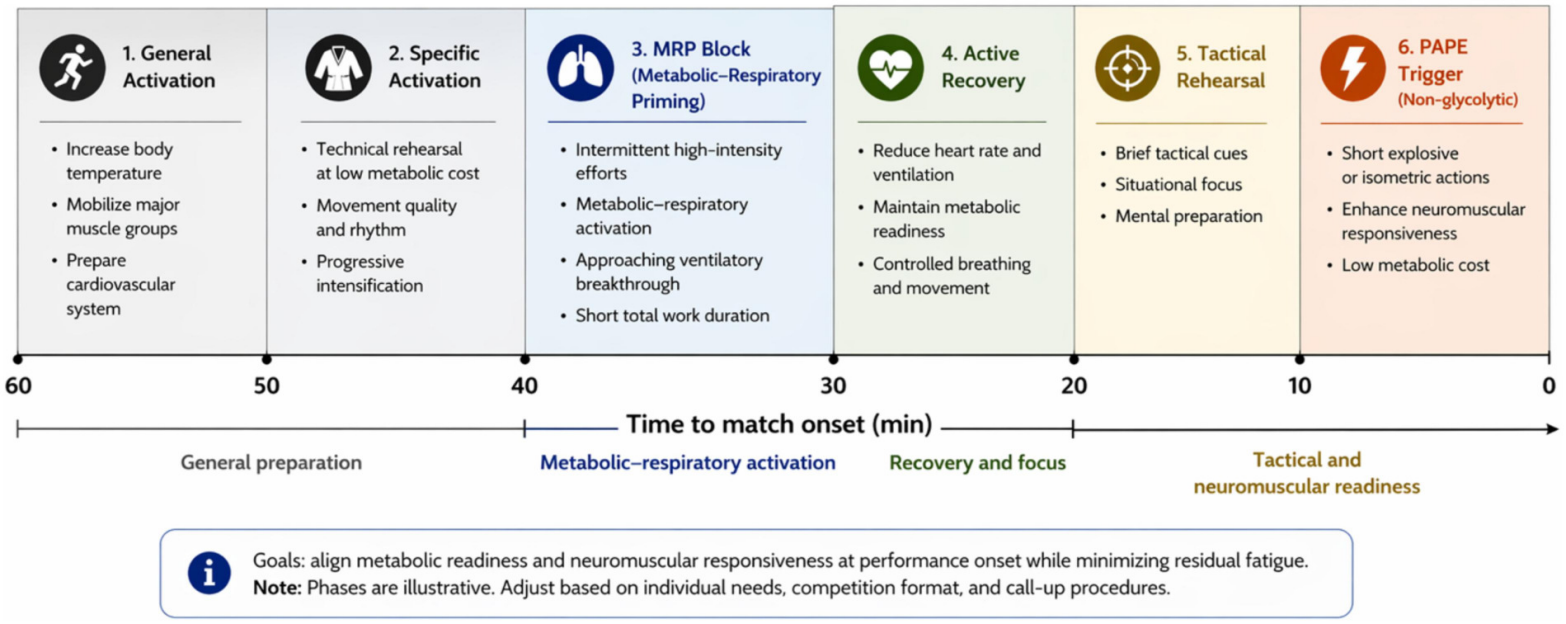
Conceptual warm-up timeline integrating Metabolic–Respiratory Priming (MRP) and PAPE.

Within a broader warm-up architecture, MRP is typically positioned as a final metabolic–respiratory phase within the warm-up sequence, preceded by general activation and technical rehearsal at low metabolic cost, and followed by controlled active recovery and brief tactical focus (Figure 2).

In elite settings, this sequence may be complemented by a short non-glycolytic neuromuscular trigger (PAPE) applied near call-up (12).

Such sequencing aims to align metabolic readiness and neuromuscular responsiveness at match onset while minimizing interference between metabolic fatigue and explosive performance.

MRP is positioned within the warm-up sequence as a metabolic–respiratory activation phase followed by controlled recovery and a brief neuromuscular trigger (PAPE), allowing metabolic readiness and neuromuscular responsiveness to converge at performance onset. The relative duration of each phase may vary depending on competition logistics and individual response.

## 6 Limitations, risks, and potential misapplications

This Perspective introduces Metabolic–Respiratory Priming (MRP) as a practitioner-derived conceptual framework grounded in established exercise physiology. Several limitations and potential risks warrant consideration.

First, this manuscript does not present original experimental data. MRP is proposed as a hypothesis-generating model informed by literature on oxygen uptake kinetics, oxygen deficit, and lactate metabolism (6, 7, 9). Accordingly, causal effects on performance cannot be inferred, and the framework should not be interpreted as evidence of efficacy. Empirical validation is required.

Second, inter-individual variability in metabolic and cardiorespiratory responses at exercise onset is substantial. Athletes differ in VO₂ kinetics, buffering capacity, lactate turnover, and recovery dynamics (7). Without appropriate calibration, excessive activation intensity or insufficient recovery following MRP may result in residual metabolic or ventilatory strain, potentially impairing early technical fluency or perceptual stability.

Third, competition ecology and logistical constraints influence practical applicability. Elite international competitions often permit structured warm-up sequencing and controlled recovery windows, whereas regional or youth tournaments may involve rapid bout turnover and unpredictable preparation time (17). Direct replication of elite-level MRP implementations may therefore be impractical or counterproductive in some contexts.

Fourth, MRP may be misapplied if conflated with neuromuscular priming strategies. Although MRP may interact beneficially with PAP/PAPE when appropriately sequenced, its primary objective is metabolic–respiratory alignment. Reducing MRP to brief explosive actions without sufficient metabolic activation would alter its intended function and may fail to address early-match metabolic lag (12).

Finally, while MRP may conceptually extend to other intermittent combat sports, rule sets, pacing structures, and technical demands differ (3). MRP should therefore be regarded as a flexible framework requiring empirical validation and contextual adaptation rather than a universally transferable prescription.

Acknowledging these limitations is essential to prevent misinterpretation and to guide future research aimed at defining dose–response characteristics, identifying population-specific responses, and evaluating performance relevance under both controlled and ecologically valid conditions.

## 7 Future research directions

With Metabolic–Respiratory Priming (MRP) formalized as a conceptual framework, several priority avenues for empirical investigation emerge.

### 7.1 Physiological validation

Controlled studies should quantify the acute effects of MRP on pulmonary oxygen uptake (VO₂) kinetics, oxygen deficit magnitude, ventilatory responses, perceived exertion, and early lactate dynamics during abrupt high-intensity exercise transitions (6, 7). Given the short temporal window in which these effects are expected, high-resolution analyses focusing on the first ∼0–180 s are warranted.

A potential validation approach could involve randomized crossover designs comparing standard warm-up strategies with MRP-based protocols. Primary outcomes may include the VO₂ on-response time constant (*τ*) and oxygen deficit magnitude, while secondary outcomes may assess early-phase blood lactate kinetics and perceptual responses.

### 7.2 Performance relevance

Research should determine whether improved metabolic–respiratory readiness translates into tactically meaningful outcomes. In judo, relevant metrics may include time to first attack, grip dominance duration, effectiveness of off-balancing actions, and tempo maintenance during the opening phase of the bout (2). Addressing these questions will likely require interdisciplinary approaches integrating exercise physiology, biomechanics, and match analysis.

### 7.3 Dose–response and population specificity

Future work should identify minimal effective doses, safety thresholds, and recovery requirements associated with MRP. Responses may vary with aerobic fitness, training age, sex, weight category, and competition structure (7, 17). Particular attention should be directed toward decay kinetics and re-activation strategies under realistic call-up uncertainty.

### 7.4 Cross-sport generalization

Comparative investigations across intermittent combat sports may clarify whether MRP reflects a judo-specific solution or a broader onset-focused preparation strategy (3).

### 7.5 Interaction with neuromuscular priming

Mechanistic research should explore how MRP and PAP/PAPE interact temporally, and whether sequencing can optimize convergence of metabolic readiness and neuromuscular responsiveness at performance onset (12, 13).

These directions may support the transition of MRP from a practitioner-derived construct to an evidence-informed preparation framework, clarifying mechanisms, boundaries, and performance relevance.

## 8 Discussion and conclusion

Combat sports present a distinctive physiological constraint: athletes must perform at competition intensity immediately at match onset, before oxidative metabolism has fully adjusted to energetic demand (6, 7). In judo, this produces a time-specific mismatch between metabolic readiness and tactical–mechanical requirements during the opening phase of the bout—an interval that may influence grip dominance, positional initiative, and match trajectory (2).

Metabolic–Respiratory Priming (MRP) is proposed as a practitioner-derived conceptual framework aimed at reducing early-match metabolic disruption by accelerating VO₂ kinetics and facilitating proactive metabolic organization prior to performance onset. MRP is not intended to replace conventional warm-up strategies, but to function as a metabolic–respiratory layer within the warm-up sequence, aligning physiological state with the demands of abrupt high-intensity combat.

Conceptually, MRP is distinct from neuromuscular priming approaches such as PAP/PAPE. Whereas neuromuscular strategies primarily target mechanical responsiveness, MRP targets metabolic–respiratory alignment and is hypothesized to exert its primary effects during the initial contest actions. When appropriately sequenced with a brief non-glycolytic PAPE stimulus, MRP may contribute to an integrated warm-up architecture in which metabolic readiness and neuromuscular responsiveness converge at performance onset without imposing excessive residual fatigue (12, 13).

By formalizing MRP, this Perspective aims to bridge applied high-performance practice and contemporary exercise physiology through a clearly defined, testable framework. Further empirical research is required to quantify underlying mechanisms, define dose–response characteristics, and evaluate relevance across competitive contexts.

## Data Availability

The original contributions presented in the study are included in the article/Supplementary Material, further inquiries can be directed to the corresponding author.
